# Construction of Potential Gene Expression and Regulation Networks in Prostate Cancer Using Bioinformatics Tools

**DOI:** 10.1155/2021/8846951

**Published:** 2021-08-31

**Authors:** Heyu Liu, Lirong Li, Yuan Fan, Yaping Lu, Changhong Zhu, Wei Xia

**Affiliations:** ^1^Institute of Reproductive Health, Tongji Medical College, Huazhong University of Science and Technology, Wuhan 430030, China; ^2^Department of Urology, Union Hospital, Tongji Medical College, Huazhong University of Science and Technology, Wuhan 430030, China; ^3^Sinopharm Genomics Technology Co., Ltd., Wuhan 430030, China

## Abstract

**Objective:**

To identify the key genes involved in prostate cancer and their regulatory network.

**Methods:**

The dataset of mRNA/miRNA transcriptome sequencing was downloaded from The Cancer Genome Atlas/the Gene Expression Omnibus database for analysis. The “edgeR” package in the R environment was used to normalize and analyze differentially expressed genes (DEGs) and miRNAs (DEmiRNAs). First, the PANTHER online tool was used to analyze the function enrichment of DEGs. Next, a protein-protein interaction (PPI) network was constructed using STRING and Cytoscape tools. Finally, miRNA-gene regulatory networks were constructed using the miRTarBase.

**Results:**

We identified 4339 important DEGs, of which 2145 were upregulated (Up-DEGs) and 2194 were downregulated (Down-DEGs). Functional enrichment analysis showed that the Up-DEGs were related to the immune system and the cell cycle in prostate cancer, whereas the Down-DEGs were related to the nucleic acid metabolic process and metabolism pathways. Twelve core protein clusters were found in the PPI network. Further, the constructed miRNA-gene interaction network showed that 11 downregulated miRNAs regulated 16 Up-DEGs and 22 upregulated miRNAs regulated 22 Down-DEGs.

**Conclusion:**

We identified 4339 genes and 70 miRNAs that may be involved in immune response, cell cycle, and other key pathways of the prostate cancer regulatory network. Genes such as BUB1B, ANX1A1, F5, HTR4, and MUC4 can be used as biomarkers to assist in the diagnosis and prognosis of prostate cancer.

## 1. Introduction

Prostate cancer (PCa) is one of the most common malignant tumors in urology. Its incidence has been increasing in recent years, and it has now become the leading cause of cancer-related deaths among middle-aged men [1]. Androgen deprivation therapy using surgical or chemical castration is the standard treatment for all stages of PCa [2]. However, patients ultimately tend to develop castration-resistant PCa, which requires further treatment. The treatment of PCa is limited by the low selectivity of medication and drug resistance encountered in all radiotherapy, chemotherapy, and immunotherapy. Thus, the reduction of multidrug resistance and identification of a clear molecular target would significantly improve the efficacy of therapeutic interventions for PCa. With the development and clinical application of molecule-targeted drugs, the molecule-targeted treatment of tumors has been widely accepted. However, there is currently a lack of precise and effective indicators to predict the efficacy of chemotherapy and targeted drug therapy. Therefore, there is an urgent need to find new indicators to indicate the use of correct drugs and improve patient survival and quality of life [3]. These new indicators or tumor molecular markers would be helpful in the diagnostic and prognostic evaluation of PCa.

With the development of high-throughput gene chip and sequencing technology, it is possible to rapidly study the gene expression profile of PCa, thereby identifying the gene expression and key gene expression changes in PCa tissues and cells under specific conditions. Bioinformatics involves gene chip data analyses. It uses sequence alignment, statistical analysis, visual mapping, biological clustering, biological molecular network, and pathway analysis to mine the massive and complex bioinformatics data generated by gene chip technology to enable more systematic study and comprehensive treatment of diseases [4]. In recent years, large-scale genome sequencing and gene chip detection approaches have been used in cancer research. The Cancer Genome Atlas (TCGA) database contains the global gene chip dataset. As the largest cancer gene information database available at present, the TCGA database includes rich and standardized clinical data on many cancer types and multiple groups, including data on gene expression, miRNA expression, copy number variation, DNA methylation, and single nucleotide polymorphism, based on large sample sizes for each cancer type. Thus, this database can be used for the search for cancer biomarkers using bioinformatics tools.

miRNAs are evolutionarily conserved short (approximately 18–22 nucleotides long) noncoding single-stranded RNA molecules that function as posttranscriptional gene regulators [5]. A large body of evidence has proven that the occurrence and development of cancer is often accompanied by the abnormal expression of some miRNAs [6]. Studies on lung cancer and breast cancer have shown that miRNAs can be used as biological targets for cancer treatment [7, 8]. Therefore, it is meaningful to use miRNAs as biomarkers for the early diagnosis and prognosis of cancer, but this use is limited as several functions and biological processes of miRNAs remain unidentified. In this study, differentially expressed genes (DEGs)/miRNAs were extracted from the microarray transcriptome data of PCa in TCGA/the Gene Expression Omnibus (GEO) database. Physiological functions and signal transduction pathways related to the DEGs were then obtained by Gene Ontology (GO) enrichment and Kyoto Encyclopedia of Genes and Genomes (KEGG) pathway analyses. Further, the protein-protein interaction (PPI) network and prostate-specific gene coexpression network were analyzed to identify the core protein clusters and key genes. Finally, the miRNA-gene interaction network was constructed. This process laid a foundation for the clinical diagnosis and prognosis of PCa.

## 2. Materials and Methods

### 2.1. RNA-Seq and miRNA-Seq Data

The transcriptome profiling datasets were downloaded from the GDC data portal [9]. The RNA-Seq dataset was obtained by advanced search with strings “cases.project.project_id” in [“TCGA-PRAD”], “files.analysis.workflow_type” in [“HTSeq - Counts”], and “files.data_category” in [“transcriptome profiling”]. And the miRNA-Seq dataset was obtained by advanced search with strings “cases.project.project_id” in [“TCGA-PRAD”], “files.data_category” in [“transcriptome profiling”], and “files.data_type” in [“miRNA Expression Quantification”].

Both the RNA-Seq and miRNA-Seq datasets originated from a total of 499 clinical PCa samples, including white (413 cases), black or African American (58 cases), Asian (12 cases), American Indian or Alaska native (1 case), and race not reported (14 cases) patients, and 52 normal prostate samples (race not reported). The patient age ranged from 41 to 78 years old.

For the RNA-Seq dataset, HTSeq-Count tables of the 499 tumor and 52 normal samples were merged to form a gene read count matrix. And for the miRNA-Seq dataset, the “read_count” columns in the quantification files were merged to form a miRNA count table.

### 2.2. Differential Expression Analyses

The read count matrices of genes and miRNAs were, respectively, used to call differentially expressed genes (DEGs) and differentially expressed miRNAs (DEmiRNAs) between tumor and normal samples by the Bioconductor package “edgeR” in the R software (version 4.0.2). The edgeR programs including filtering, normalization, dispersion estimating, and quasilikelihood *F*-tests were performed. The cut-off we used to pick significant DEGs and DEmiRNAs was *p* value < 0.05, false discovery rate (FDR) < 0.05, and ∣log2FC | >1. The log-fold change against log-counts per million, with DEGs or DEmiRNAs highlighted, was plotted.

### 2.3. Gene Functional Enrichment Analyses

The official Gene Ontology (GO) online tool (http://geneontology.org/) with human genes as the background was used to implement Gene Ontology enrichment. Ensemble gene lists of 2145 upregulated DEGs (Up-DEGs) and 2194 downregulated DEGs (Down-DEGs) were separately submitted to the web service powered by PANTHER. Overrepresentation tests (released 20200728) were performed with Fisher's exact test as the test type and the calculated false discovery rate as the correction method. The GO terms were sorted and filtered by FDR and fold enrichment. And the top 10 terms of the three GO domains (cellular component, biological process, and molecular function) were shown.

Kyoto Encyclopedia of Genes and Genomes (KEGG) pathway enrichment analyses were performed to reveal signaling pathways in prostate cancer. Firstly, ensemble IDs of DEGs were converted and filtered to 1481 Up-DEGs' symbols and 1961 Down-DEGs' symbols by the HGNC (HUGO Gene Nomenclature Committee) BioMart server. Then, the DEGs' symbols were called by the gene-list enrichment tool in KOBAS3.0 to do KEGG pathway enrichment with default parameters. The cut-off for significant pathways was set as corrected *p* value < 0.05, and the top 10 pathways were shown.

### 2.4. Construction of Protein-Protein Interaction (PPI) Networks

Firstly, 1481 Up-DEGs' symbols and 1961 Down-DEGs' symbols were separately input to the Search Tool for the Retrieval of Interacting Genes/Proteins (STRING) online tool [10] to build PPI networks, with “the minimum required interaction score” set as “highest confidence (0.900)” and the “hide disconnected nodes in the network” option was checked. And the tabular text output PPI files were exported.

Secondly, the PPI files were imported into the Cytoscape 3.8.2 software [11]. The MCODE application was used to find clusters (highly interconnected areas) in the network, and the score of key PPI nodes was calculated using the *k*-core decomposition algorithm. The cluster finding parameters were node score cutoff, 0.2; haircut, true; fluff, false; *k*-core, 7; and max. depth from seed, 100. The score of nodes reflected the density of the nodes and the surrounding nodes. Linked proteins had the same score and formed core protein clusters.

Finally, to depict the PPI networks of the core protein clusters, yFiles Layout Algorithms in Cytoscape applications were used.

### 2.5. Profiling of miRNAs and Gene Regulation Networks

50 upregulated DEmiRNAs (Up-DEmiRNAs) and 20 downregulated DEmiRNAs (Down-DEmiRNAs) were compared to PCa-related miRNAs in miRCancer (miRNA Cancer Association Database), and a Venn diagram was drawn.

The miRTarBase provides information about experimentally validated miRNA-target gene interactions [12, 13]. To obtain miRNA-gene interactions in PCa regulation, the 91 Up-DEGs from five upregulated and 137 Down-DEGs from seven downregulated core protein clusters were used to bait the corresponding miRNA regulators verified by comprehensive experiments.

To increase the reliability of miRNA-gene interactions in PCa, we selected the DEmiRNAs and the corresponding DEGs as high-confident regulation pairs. The experimentally validated high-confident regulation networks were constructed and displayed using the Cytoscape 3.8.2 software.

## 3. Results

### 3.1. Identification of DEGs in Prostate Cancer Response

To know how genes respond in prostate cancer, we collected RNA-Seq datasets from the TCGA-PRAD project, including 449 tumor samples and 52 normal samples, and performed transcriptome profiling.

**Figure 1:**
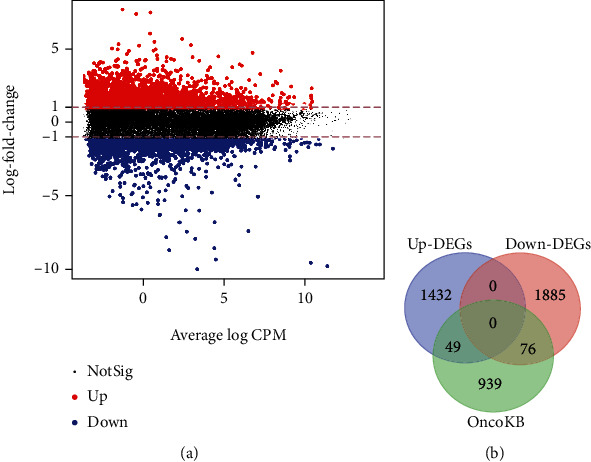
Differentially expressed genes (DEGs) between PCa and normal samples. (a) The MA plot of DEGs. (b) Venn diagram of DEGs overlapped with OncoKB cancer genes.

Differential expression analyses uncovered DEGs either upregulated or downregulated in comparison between tumor and normal. In total, 4339 DEGs were identified, and the screening criteria were (1) ∣logFC | >1, (2) *p* < 0.05, and (3) FDR < 0.05. The MA plot gives a quick overview of the 2145 upregulated DEGs (Up-DEGs) and 2194 downregulated DEGs (Down-DEGs) (Figure 1(a)).

The ensemble IDs of DEGs were converted and filtered to 1481 Up-DEGs' symbols and 1961 Down-DEGs' symbols, which were then compared with the OncoKB cancer gene list. 125 DEGs identified in this study were also found in OncoKB, but we also detected a large proportion of DEGs (96.4%, 3317/3442) that have potential to be actionable genes in prostate cancer (Figure 1(b)).

### 3.2. Enrichment of Gene Functions in Prostate Cancer

**Figure 2:**
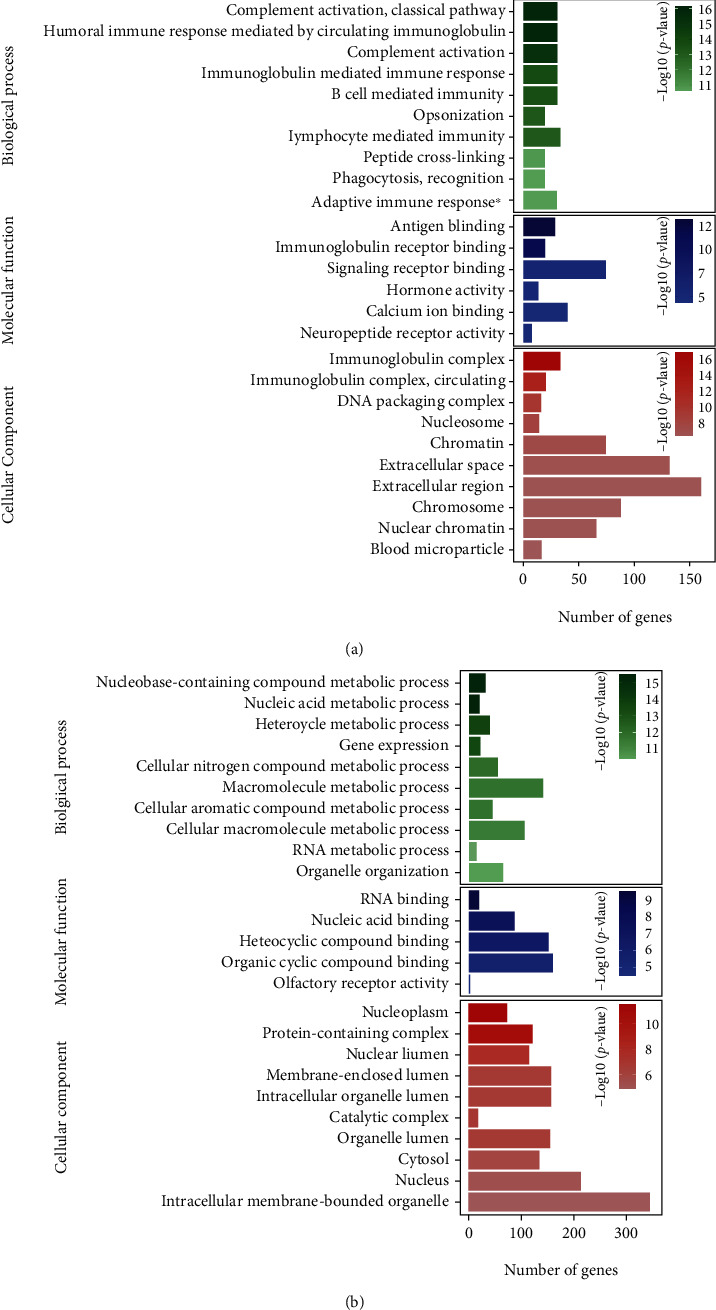
Enrichment of Gene Ontologies (GO) of Up-DEGs and Down-DEGs: (a) GO enrichment of Up-DEGs; (b) GO enrichment of Down-DEGs. The top 10 terms in the three GO domains (biological process, molecular function, and cellular component) are shown.

To reveal effective biological functions in prostate cancer, Gene Ontology (GO) enrichment analyses of DEGs are conducted. The GO enrichment analysis of the 2145 Up-DEGs showed that in biological processes, they were mainly enriched in complement activation, classical pathway, humoral immune response mediated by circulating immunoglobulin, complement activation, and immunoglobulin-mediated immune response; in molecular functions, mainly in antigen binding, immunoglobulin receptor binding, signaling receptor binding, and hormone activity; and in cellular components, mainly in immunoglobulin complex, DNA packaging complex, nucleosome, and chromatin (Figure 2(a)). The GO enrichment analysis of the 2194 Down-DEGs showed that in biological processes, they were mainly enriched in nucleobase-containing compound metabolic process, nucleic acid metabolic process, heterocycle metabolic process, and gene expression; in molecular functions, mainly in RNA binding, nucleic acid binding, heterocyclic compound binding, and organic cyclic compound binding; and in cellular components, mainly in nucleoplasm, protein-containing complex, nuclear lumen, and membrane-enclosed lumen (Figure 2(b)).

To profile prostate cancer-responsive mechanisms, enrichment analyses of biological pathways defined by Kyoto Encyclopedia of Genes and Genomes (KEGG) were carried out. Most of the Up-DEGs were significantly enriched in the pathways termed as “neuroactive ligand-receptor interaction,” “cell cycle,” “complement and coagulation cascades,” “oocyte meiosis,” “maturity onset diabetes of the young,” “nicotine addiction,” “linoleic acid metabolism,” and “bile secretion” (Supplementary Figure 1A), whereas the Down-DEGs were mainly involved in “calcium signaling pathway,” “metabolic pathways,” “neuroactive ligand-receptor interaction,” “focal adhesion,” “cAMP signaling pathway,” “arrhythmogenic right ventricular cardiomyopathy (ARVC),” “dilated cardiomyopathy (DCM),” “hypertrophic cardiomyopathy (HCM),” “gastric acid secretion,” and “PI3K-Akt signaling pathway” (Supplementary Figure 1B).

These results suggest the importance of these pathways in PCa medical mechanisms.

### 3.3. Core Protein-Protein Interaction (PPI) Networks in Prostate Cancer

To do further functional research of the DEGs, the STRING database providing functional association networks was retrieved. First, the identified Up-DEGs and Down-DEGs were, respectively, submitted to the STRING database to construct PPI networks. And “the minimum required interaction score” was set to the “highest confidence (>0.9)” to filter high-confident interactions. Next, to discover core protein clusters hidden under the huge networks, MCODE clustering algorithms in Cytoscape 3.8.2 were applied. The score of key PPI nodes was calculated using the *k*-core decomposition algorithm, and the functional clusters with scores ≥ 7, referred to as the “core protein clusters,” were screened out.

**Figure 3:**
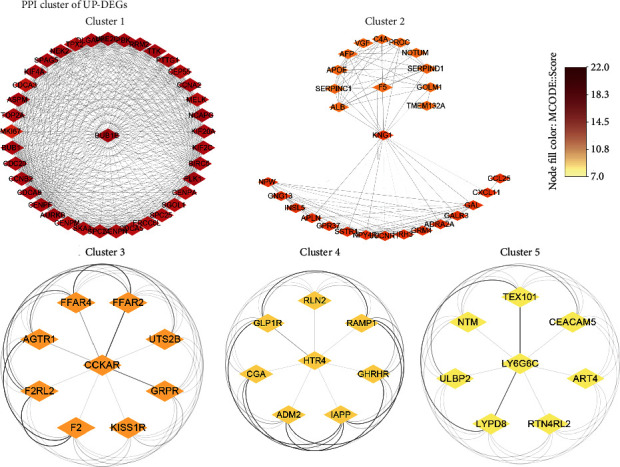
Core protein-protein interaction (PPI) networks of Up-DEGs. The network nodes are proteins. The edges represent the predicted functional associations. The node fill color mapped the MCODE score, reflecting the density of the nodes and the surrounding nodes. The edge transparency represents the combined interaction score between two nodes.

In the PPI network of the Up-DEGs, there are 907 nodes and 1307 edges retained, and the average node degree is 2.88. The expected number of edges is 497, and the network has significantly more interactions than expected (the PPI enrichment *p* value < 1.0*e*-16) (not shown). Finally, five core protein clusters of Up-DEGs were constructed (Figure 3). Cluster 1 has the maximum score 27.676, with 38 nodes and 512 edges, including known cancer-related genes, such as BUB1 (mitotic checkpoint serine/threonine-protein kinase BUB1), CDC20 (cell division cycle protein 20 homolog), and PLK1 (serine/threonine-protein kinase PLK1). Cluster 2 has 28 nodes and 198 edges, including proteins in the coagulation system, such as KNG1 (kininogen-1) and F5 (pronounced factor five). Cluster 3 has 9 nodes and 36 edges, including the GRPR (gastrin-releasing peptide receptor) which is known to be expressed in numerous cancers. Furthermore, there are 8 nodes and 8 edges in both cluster 4 and cluster 5.

**Figure 4:**
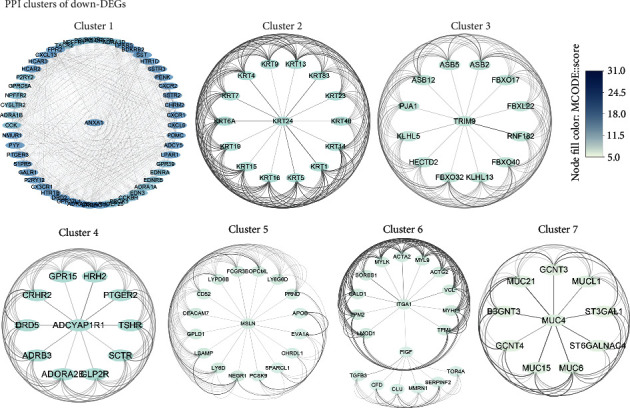
Core protein-protein interaction (PPI) networks of Down-DEGs. The network nodes are proteins. The edges represent the predicted functional associations. The node fill color mapped the MCODE score, reflecting the density of the nodes and the surrounding nodes. The edge transparency represents the combined interaction score between two nodes.

In the PPI network of the Down-DEGs, 1668 nodes and 2524 edges were identified, and the average node degree is 3.03. The expected number of edges is 1389, and the network has significantly more interactions than expected (the PPI enrichment *p* value < 1.0*e*-16) (not shown). Finally, seven core protein clusters of Down-DEGs were constructed (Figure 4). Cluster 1 has the maximum score 32.549, with 52 nodes and 830 edges, including known cancer-related genes, such as ANXA1 (annexin A1) and SSTR2 (somatostatin receptor type 2). Cluster 2 has 15 nodes and 105 edges, including KRT (keratin) family proteins whose expression is helpful in determining the epithelial origin in anaplastic cancers. Cluster 3 has 13 nodes and 78 edges, including the FBXO32 (F-box only protein 32) which was reported to be associated with tumorigenesis. Further, there are 11 nodes and 55 edges in cluster 4, 17 nodes and 81 edges in cluster 5, 19 nodes and 88 edges in cluster 6, and 10 nodes and 40 edges in cluster 7.

The above results suggest that all of these PPI interactions in the core protein clusters play essential roles in prostate cancer regulation networks and deserved further research.

### 3.4. Well-Grounded miRNA-Gene Regulation Networks in PCa

To explore how miRNAs respond in prostate cancer, we collected miRNA-Seq datasets from the TCGA-PRAD project, including 449 tumor samples and 52 normal samples, and performed miRNA-Seq analyses.

**Figure 5:**
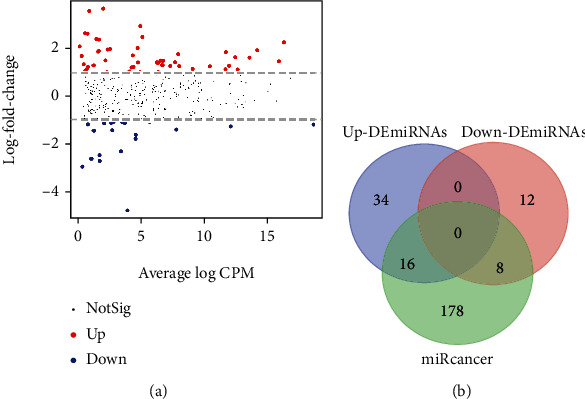
Differentially expressed miRNAs (DEmiRNAs) between PCa and normal samples. (a) The MA plot of DEmiRNAs. (b) Venn diagram of DEmiRNAs overlapped with PCa-related miRNAs in miRCancer.

Firstly, differential expression analyses uncovered DEmiRNAs either upregulated or downregulated in comparison between tumor and normal. In total, 70 DEmiRNAs were identified, and the screening criteria were (1) ∣logFC | >1, (2) *p* < 0.05, and (3) FDR < 0.05. The MA plot gives a quick overview of the 50 upregulated DEmiRNAs (Up-DEmiRNAs) and 20 downregulated DEmiRNAs (Down-DEmiRNAs) (Figure 5(a)).

Compared to PCa-related miRNAs in miRCancer (miRNA Cancer Association Database), 24 DEmiRNAs identified in this study were also found in miRCancer, but we also detected more than half of DEmiRNAs (65.7%, 46/70) that have potential to be actionable miRNAs in prostate cancer (Figure 5(b)).

Subsequently, the DEGs in the five upregulated core protein clusters and the seven downregulated core protein clusters were uploaded as “seeds” to the miRTarBase (experimentally validated miRNA-target interaction database), and the miRNA-DEG interaction network verified by comprehensive experiments was obtained. The results showed that in the core protein cluster, 91 Up-DEGs interacted with 829 miRNAs and 137 Down-DEGs interacted with 791 miRNAs.

**Figure 6:**
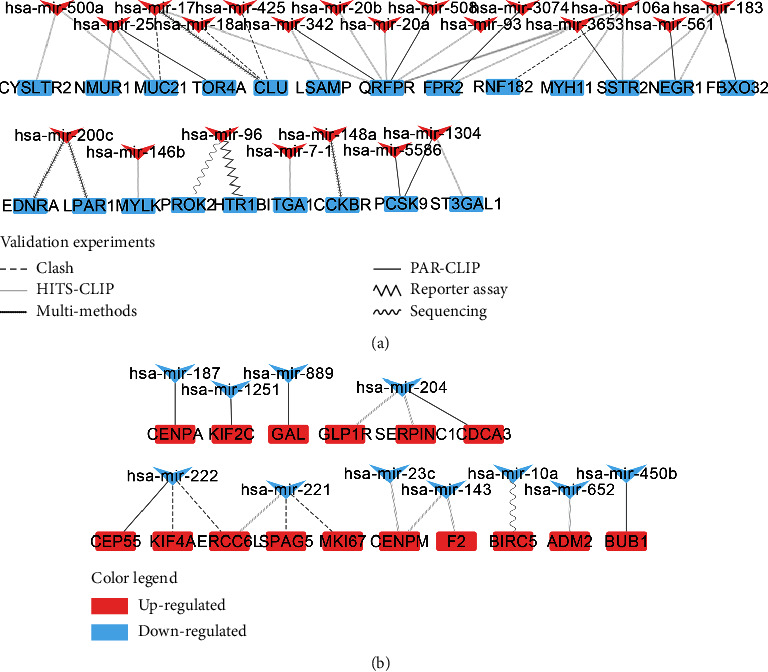
Experimentally validated DEmiRNA-DEG interaction networks: (a) Up-DEmiRNAs targeted Down-DEGs; (b) Down-DEmiRNAs targeted Up-DEGs. Line types indicate validation experiments of the interactions. “Multi-methods” includes luciferase reporter assay, qRT-PCR, and Western blot.

Lastly, miRNAs are noncoding single-stranded small molecular RNAs that are highly conserved in evolution and regulate gene expression through translational inhibition. Therefore, the 791 miRNAs that were found to interact with the 137 downregulated “seeds” were crossed with the 50 Up-DEmiRNAs; this revealed 22 Up-DEmiRNAs (hsa-mir-500a, hsa-mir-17, hsa-mir-425, hsa-mir-20b, hsa-mir-508, hsa-mir-3074, hsa-mir-106a, hsa-mir-183, hsa-mir-25, hsa-mir-18a, hsa-mir-342, hsa-mir-20a, hsa-mir-93, hsa-mir-3653, hsa-mir-561, hsa-mir-200c, has-mir-96, has-mir-148a, has-mir-1304, has-mir-146b, has-mir-7-1, and has-mir-5586) that could predict the gene expression regulation in PCa (Figure 6(a)). Next, the 829 miRNAs that were found to interact with the 91 upregulated “seeds” were crossed with the 20 Down-DEmiRNAs; this revealed 11 Down-DEmiRNAs (hsa-mir-187, hsa-mir-1251, hsa-mir-889, hsa-mir-204, hsa-mir-222, hsa-mir-221, hsa-mir-23c, hsa-mir-143, hsa-mir-10a, hsa-mir-652, and hsa-mir-450b) that could predict the gene expression regulation in PCa (Figure 6(b)).

Taken together, we constructed the experimentally validated high-confident regulation networks of the DEmiRNAs and the corresponding DEGs in PCa, which indicate that these miRNA-Gene interactions play essential roles in PCa molecular regulation.

## 4. Discussion

In this study, we attempted to identify tumor microenvironment-related genes/miRNAs from the TCGA database that contribute to PCa occurrence and development. First, there were 2145 upregulated genes and 2194 downregulated DEGs between PCa and normal samples. Next, the DEGs, were subsequently subjected to GO and KEGG pathway enrichment analysis, which showed that these DEGs were significantly enriched in the functional modules and biological process of cancer development, and indicated some significant characteristics of PCa, such as hyperactivity of immune response [14], hormone activity, diabetes [15, 16], and nicotine addiction [17]. Finally, the results of PPI network analysis and prostate tissue-specific gene coexpression network analysis revealed that six upregulated genes (BUB1B, F5, KNG1, CCKAR, HTR4, and LY6G6C) and eight downregulated genes (ANXA1, KRT24, TRIM9, ADCYAP1R1, MSLN, ITGA1, FIGF, and MUC4) were present as the core genes in the prostate tissue-specific gene coexpression network.

These genes play an important role in various human cancers, including prostate cancer. For example, Rajan et al. identified seven hub genes (ADAM7, fam72b, BUB1B, ccnb1, ccnb2, TTK, and cdk172) related to cell cycle in prostate biopsy tissues before and after docetaxel chemotherapy and androgen deprivation therapy in patients with advanced hormone-naive prostate cancer [18]. BUB1B also had differential expressions in our results. BUB1B is a key mitotic checkpoint kinase. Ding et al. identified BUB1B as the top-scoring kinase by RNA interference and bioinformatics analysis, which can monitor proper spindle microtubule attachment to the kinetochore, and it is knocked down inducing mitotic catastrophe and cell death in glioblastoma [19, 20]. The above research suggests that BUB1B has potential to be a novel antimitotic target in some cancers, including prostate cancer.

For another example, our study found that the expression of ANXIA1 is downregulated in prostate cancer, and the results are also proven in other literatures [21–23], which occurs in the early stage of cancer or intraepithelial tumor transformation of prostate cancer and becomes more prominent with the development of cancer. Inokuchi et al. proved that reducing the expression of ANXA1 can enhance the invasion of prostate cancer tumor by upregulating the expression and activity of IL-6 [24]. Therefore, the loss of ANXA1 may be a useful marker for the development and progression of prostate cancer. However, some studies have proven that the expression of ANXA1 is negatively correlated with androgen receptor (AR), and the expression of ANXA1 increases after AR knockdown or AR antagonists are used, which accelerates the invasion and metastasis of advanced PCa [25, 26]. ANXA1 may act as a tumor inhibitor in the early stage of cancer, but in the late stage of cancer, it may play the opposite role. To sum up, as a “double-edged sword,” the clinical research and treatment of using ANXA1 as tumor inhibitors should be cautious and limited.

The F5 gene, which is the most common genetic coagulation factor mutation, also increases the risk of thrombosis. Garber et al. found that F5 gene variation was associated with breast cancer. The F5 expression was enriched in breast cancer and was associated with overall survival [27]; moreover, the F5 gene was associated with the risk of thrombosis in cancer patients [28]. F5 is also associated with the risk of thrombosis in patients with metastatic androgen-dependent prostate cancer who undergo diethylstilbestrol and docetaxel chemotherapy [29]. This provides an interesting direction for further research to strengthen the relationship between cancer and coagulation.

MUC4 usually plays an important role in the pathogenesis of pancreatic, ovarian, and breast malignancies [30–32]. Through abnormal overexpression, MUC4 can interact with HER2 (a ligand-dependent receptor tyrosine kinase) physically and phosphorylated activate and stabilize HER2 to promote tumor invasion and metastasis. Our study has proven that MUC4 is downregulated in prostate cancer tissues, like other literatures [33, 34]. In line with our results, Dizeyi et al. found that the HTR4 expression was upregulated in prostate cancer [35]. They found that HTR4 is associated with the late progression of hormone refractory prostate cancer, possibly due to the paracrine/autocrine mechanism of HTR4-induced hormones or growth factors, and HTR4 is also associated with estrogen receptor *α* and estrogen receptor *β*. The overexpression of the receptor of the neuroendocrine cell product may be related to the occurrence of hormone refractory prostate cancer, which provides a new direction for the trigonometric relationship between cancer neuroendocrine sex hormones. Our study first described the upregulation of KNG1 and CCKAR in prostate cancer. Previous studies have described the presence of KNG1 and CCKAR as biomarkers of various types of cancer, such as thyroid cancer [36, 37], liver cancer [38], ovarian cancer [39], and cholangiocarcinoma [40]. In different stages of PCa development and progression, especially in the process of hormone-sensitive PCa progressing to castration-resistant PCa, the proteomic alterations and transcriptomic data have significant differences in changes [41]. By analyzing the microarray-based profiling data of isogenic prostate cancer xenograft models published by Chen et al. [42], we found that the differentially expressed genes of hormone-sensitive PCa compared with castration-resistant PCa included BUB1B, ADCYAP1R1, HTR4, and LY6G6C, which was also proposed in our study. These results indicate that the core of this study has the potential to become a new biomarker of prostate cancer, especially for the prognosis evaluation of castration-resistant PCa.

In addition, our study also reported the full expression of microRNA in prostate cancer and predicted the microRNA/mRNA interaction network in a very reliable way. In this study, several microRNAs were first proposed to upregulate or downregulate differential expression in prostate cancer tissues. Some of them have been reported in the literature. For instance, Schaefer et al. found that 15 differentially expressed microRNAs were related to the diagnosis and prognosis of prostate cancer [43], of which the upregulated hsa-mir-183 and downregulated hsa-miR-222 overlap our results. hsa-mir-25 is related to the invasion of prostate cancer and may be a signaling mechanism of aurora kinase A or integrin [44]. Yang et al. found that hsa-mir-93 can act as a tumor promoter through the regulatory axis Dab2/AKT/ERK1/2 [45]. hsa-mir-200c can reverse the epithelial stromal transformation of prostate cancer [46]. hsa-mir-204 has been widely studied in prostate cancer, which is negatively related to the expression of UCA1 and plays a role in tumor metastasis and sensitivity to chemotherapy [47]. The functions of these miRNAs in prostate cancer deserve further investigation.

## 5. Conclusions

We constructed a series of functional networks centered on core genes involved in PCa. These networks provide new ideas for future research on the occurrence, development, and metastasis of PCa and also indicate potential new targets and biomarkers for its clinical treatment and diagnosis, respectively.

## Data Availability

The datasets used and/or analyzed during the current study are available from the corresponding authors on reasonable request.

## Supplementary Materials

Supplementary Materials Figure 1: KEGG pathway of DEGs in prostate cancer response mechanisms.
