# Supplementary figures and images for: Construction of Potential Gene Expression and Regulation Networks in Prostate Cancer Using Bioinformatics Tools
Source: Oxid Med Cell Longev. 2021 Aug 31;2021:8846951. doi: 10.1155/2021/8846951 (PMC8426106; doi:10.1155/2021/8846951)

**A**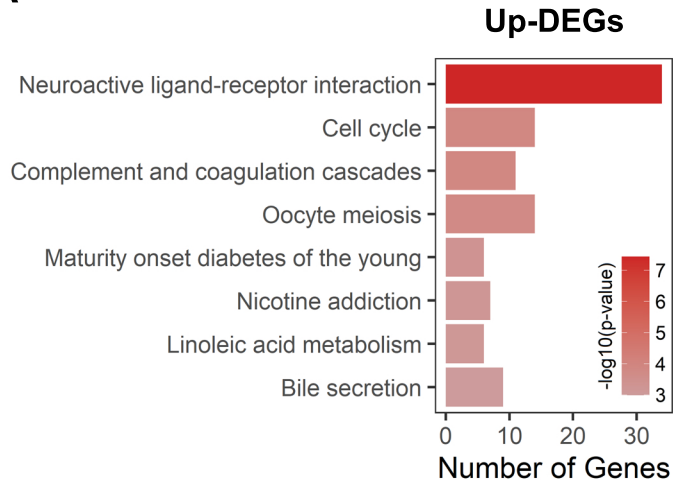**B**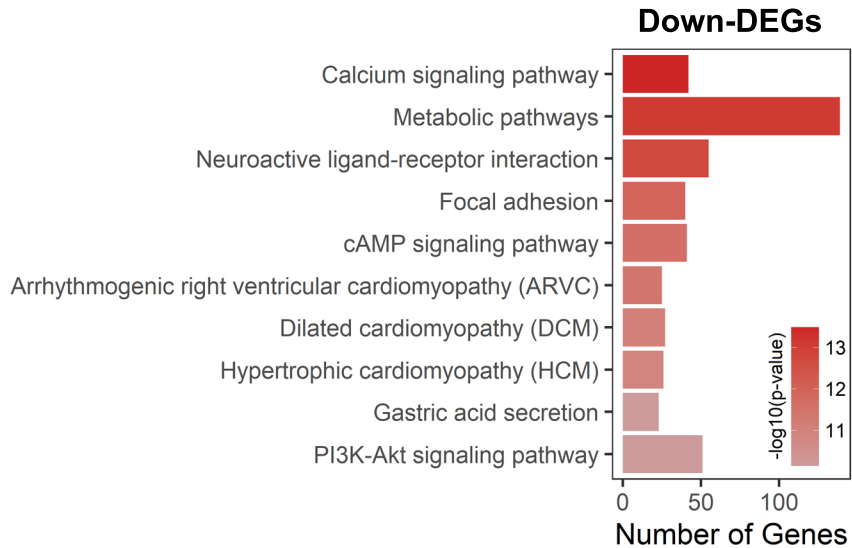

Supplement: Supplementary Materials — Figure 1: KEGG pathway of DEGs in prostate cancer response mechanisms. [file 8846951.f1.pdf]
